# Association Between the Accumulation of Pentosidine at the Sciatic Nerve and Cutaneous Hindpaw Hypersensitivity in a Rat Ovariectomy Model

**DOI:** 10.7759/cureus.21059

**Published:** 2022-01-09

**Authors:** Tomotaka Umimura, Yawara Eguchi, Sumihisa Orita, Kazuhide Inage, Koki Abe, Masahiro Inoue, Hideyuki Kinoshita, Masaki Norimoto, Masashi Sato, Takashi Sato, Masahiro Suzuki, Satoshi Maki, Takeo Furuya, Seiji Ohtori

**Affiliations:** 1 Orthopaedic Surgery, Chiba University, Chiba, JPN; 2 Center for Frontier Medical Engineering, Chiba University, Chiba, JPN

**Keywords:** advanced glycation end-products, rats, sciatic nerve, ovariectomy, cutaneous, pain, hypersensitivity, pentosidine

## Abstract

Introduction

Advanced glycation end-products (AGEs) have the potential to serve as biomarkers of aging and metabolic diseases; however, how their expression relates to clinical symptoms is not well defined. In this study, we sought to determine whether the accumulation of pentosidine, one type of AGE, at the peripheral nerve is associated with cutaneous pain or hypersensitivity caused by ovariectomy (OVX).

Methods

We assigned 12-week-old female Sprague Dawley rats into either the OVX group (n = 6) or the sham group (n = 6). Cutaneous hindpaw sensitivity to mechanical stimuli was measured with von Frey filaments, using Chaplan’s adapted method, and the 50% withdrawal threshold was calculated. Then, the accumulation of pentosidine, which represents AGEs, was measured in sciatic nerve fibers after staining with an anti-pentosidine antibody.

Results

OVX rats showed significantly increased plantar hypersensitivity to mechanical stimuli compared to sham rats 8 weeks after OVX (*P* = 0.017). Pentosidine-positive sciatic nerves were detected at a higher rate in OVX rats than in sham rats (*P* = 0.035). The pentosidine positivity rate in sciatic nerve fibers showed a negative correlation with withdrawal threshold (*P* < 0.001).

Conclusions

This study showed that higher levels of pentosidine in sciatic nerve fibers are associated with higher plantar hypersensitivity. Accumulation of pentosidine at the sciatic nerve, caused by OVX, may result in cutaneous hindpaw hypersensitivity.

## Introduction

Cutaneous pain hypersensitivity and allodynia in postmenopausal females have been reported. Specifically, postmenopausal osteoporotic females may experience bone fragility-induced fractures as well as osteoporosis-related pain that is not associated with bone injury, but where the sensory nerve fibers are hypersensitized [1]. However, the mechanisms underlying this pain are unclear. Ovariectomized rodents (OVX model) are used to model postmenopausal osteoporosis. Some reports indicate that, similar to humans, OVX rodents also show cutaneous hindpaw hypersensitivity, allodynia, and deep musculoskeletal pain [2,3].

Advanced glycation end-products (AGEs) are molecules that have recently become known as potential biomarkers for aging and certain metabolic diseases. Pentosidine, one such AGE, forms when sugars are covalently bound with proteins and other molecules through non-enzymatic processes, and thus AGEs are an indicator of lifestyle-related diseases [4,5]. Estrogen deficiency caused by menopause and aging also increases oxidative stress and may result in the generation of AGEs [6]. Umimura et al. have reported that AGEs can act as clinical biomarkers for symptoms like lower extremity pain and numbness [7]. However, the exact role of AGEs in these symptoms is not fully understood.

We hypothesized that accumulation of AGEs, such as pentosidine, at the peripheral nerve is associated with cutaneous pain hypersensitivity and allodynia in postmenopausal females or the OVX model. In this study, we sought to determine whether pentosidine accumulation at the sciatic nerve is associated with cutaneous hindpaw hypersensitivity in the rat OVX model.

## Materials and methods

Animal preparation

Female Sprague Dawley rats (Japan SLC, Japan) were used for all experiments. Rats remained on a 12-hour light/dark cycle and were housed in ventilated racks. Rats were habituated to these housing conditions for 1 week prior to any experiment. Rats were randomized into one of two groups to receive either ovariectomies (OVX group; n = 6) or sham surgeries (sham group; n = 6). One rat died 3 days after ovariectomy and was excluded, and the OVX group was finally examined with five rats. All rats were evaluated for mechanical sensitivity of the hindpaw at 0, 2, 4, and 8 weeks after surgery.

All protocols for animal procedures were reviewed and approved by the ethical review committee of our institution (institutional review board [IRB] Approval code: 30-222). Animals were treated in accordance with our affiliated institution’s guidelines.

Surgical procedure

Surgeries were conducted on 12-week-old rats. We used a model of OVX-induced osteoporosis. OVX was performed by incising a 1.5 cm dorsal midline incision, and then each ovary was ligated and resected, followed by placing the uterine horns back into the body cavity. We then closed the muscle wall and skin incisions using 6-0 silk sutures. In rats that were sham-operated, ovaries were left intact after being exposed to the same procedure as for OVX.

Behavioral procedure: cutaneous plantar sensitivity to mechanical stimuli

Behavioral testing was conducted between 9:00 a.m. and 3:00 p.m. Prior to any manipulation, rats were habituated to the testing room for 60 minutes while remaining in their home cages. Then, rats were individually habituated in the testing chamber for an additional 60 minutes prior to the behavioral procedure.

We used von Frey filaments (Stoelting, Wood Dale, IL) to measure mechanical sensitivity; filaments were applied for 4 seconds or until paw withdrawal to the left hindpaw plantar surface. To obtain the 50% threshold for withdrawal (g), we used an adapted up-and-down technique from Chaplan’s method [8].

The stimulus intensities were 10 g, 15 g, 26 g, 60 g, and 100 g, which corresponded, respectively, to filament numbers 5.07, 5.18, 5.46, 5.88, and 6.10.

Pentosidine staining of the sciatic nerve

Eight weeks after surgery, rats were sacrificed and the sciatic nerve was taken from the left hindlimb for immunohistochemical studies (Table 1). Nerve samples were fixed by 4% paraformaldehyde. They were later stained for pentosidine using an anti-pentosidine mouse monoclonal antibody (clone: PEN-12; KH012, Trans Genic Inc., Fukuoka, Japan) and a horseradish peroxidase (HRP)-conjugated anti-mouse IgG goat antibody (#424134, Nichirei Corporation, Tokyo, Japan), and then visualized with 3,3'-diaminobenzidine-tetrahydrochloride (DAB.4HCl).

**Table 1:** Immunohistochemical staining protocol for pentosidine Pentosidine staining of the sciatic nerve. RT: room temperature

Step	Process	Used antibodies or reagents	Temp.	Time
1	Deparaffinization	Xylene and 100%-80% downgraded ethanol series	RT	
2	Antigen retrieval	(A) 0.1% trypsin / (B) Not performed	37℃	15 min
3	Endogenous enzyme blocking	3% hydrogen peroxide (H_2_O_2_)	RT	15 min
4	Washing	50 mM phosphate buffer (pH 7.6)	RT	3 min ×3
5	Non-specific protein blocking	Blocking One reagent (#03953-95, Nacalai Tesque, Kyoto, Japan)	RT	10 min
6	Primary antibody reaction	Anti-pentosidine mouse monoclonal antibody, clone: PEN-12 (KH012, Trans Genic, Fukuoka, Japan) 1: 1000	4℃	overnight
7	Washing	50 mM Phosphate buffer (pH 7.6)	RT	3 min ×3
8	Secondary antibody reaction	HRP-conjugated anti-mouse IgG goat antibody (#424134, Nichirei Corporation, Tokyo, Japan)	RT	30 min
9	Washing	50 mM phosphate buffer (pH 7.6)	RT	3 min ×3
10	Visualization	3,3'-diaminobenzidine‐tetrahydrochloride (DAB.4HCl)	RT	1 min
11	Washing	Distilled water	RT	
12	Counterstain	Mayer’s hematoxylin	RT	10 sec
13	Dehydration	80%-100% upgraded ethanol series and xylene	RT	
14	Mounting	Entellan New (#107961, Merck, Darmstadt, Germany)	RT	

Specimen slides were examined under a 400× microscope and nerve fibers were counted. We calculated the ratio of the number of pentosidine-stained nerve fibers to the total number of nerve fibers as pentosidine positivity rate. Measurements were taken at three locations per slide, and the mean values were calculated.

Data analyses

We calculated the correlation between the 50% pain threshold by the von Frey test and the pentosidine positivity rate in sciatic nerve fibers. Data was plotted as means ± standard error. The number of rats were indicated by n. Statistical significance was considered when 𝑃 < 0.05. The comparison between OVX group and sham group was analyzed using Mann-Whitney U test, at 0, 2, 4, and 8 weeks after surgery.

## Results

Effects of OVX on cutaneous plantar hypersensitivity

**Figure 1:**
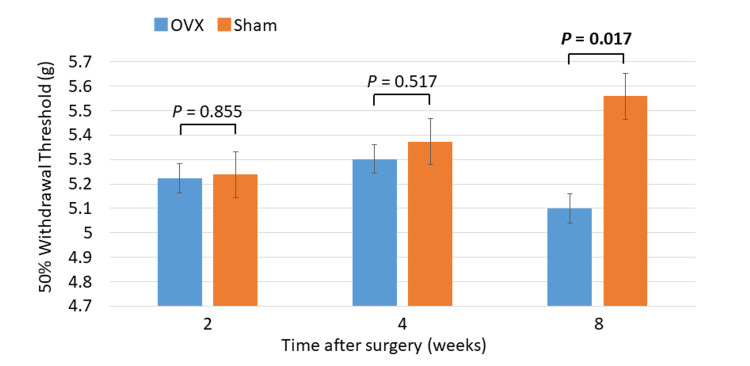
Hindpaw hypersensitivity using the von Frey test The ovariectomy group (OVX) shows significantly increased plantar hypersensitivity to mechanical stimuli, as shown by the withdrawal threshold, compared to sham controls 8 weeks after ovariectomy (*P* = 0.017; Mann–Whitney U test).

There was no significant difference between the OVX and sham groups in mechanical sensitivity by von Frey test at 2 and 4 weeks after OVX. Conversely, the OVX group showed a significantly increased plantar hypersensitivity to mechanical stimuli compared to sham controls 8 weeks after OVX (*P* = 0.017; Figure 1).

Immunohistochemical studies

**Figure 2:**
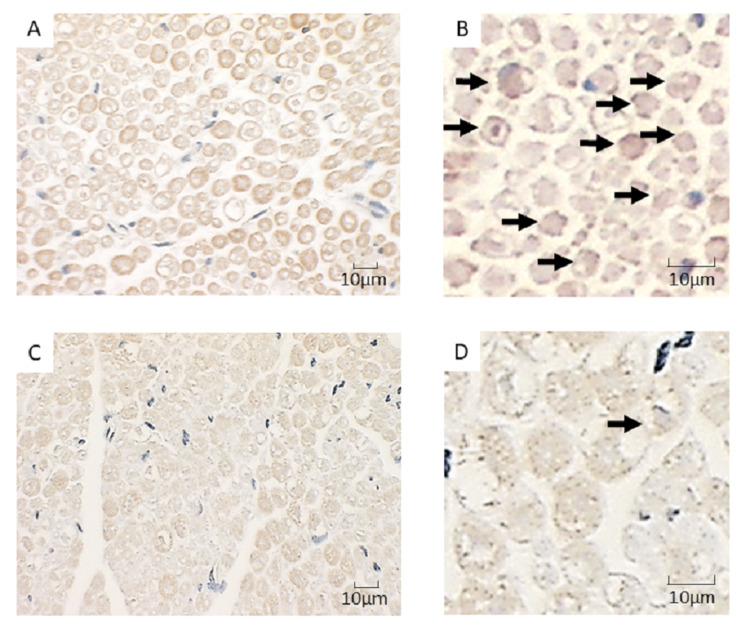
Representative images of pentosidine staining of sciatic nerves from ovariectomized (OVX) and sham rats Pentosidine staining (brown color) is visualized with the anti-pentosidine antibody (Arrows). (A, B) Brown pentosidine staining is shown by an increased accumulation of pentosidine in sciatic nerves of OVX rats. (C, D) The sciatic nerves of sham rats have fewer pentosidine-positive nerves than the OVX group.

**Figure 3:**
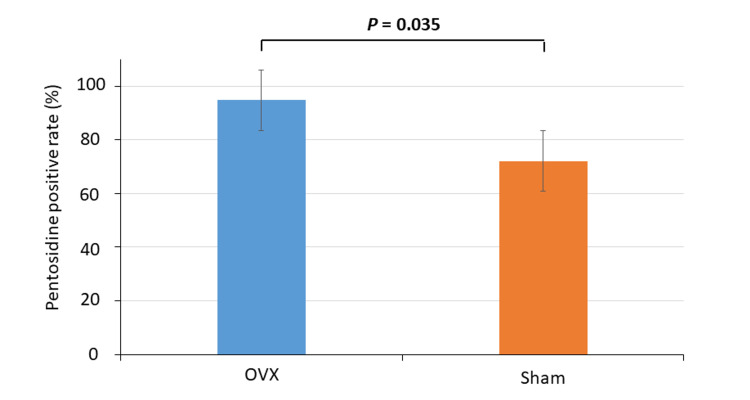
Pentosidine positivity rate of sciatic nerve fibers Pentosidine-positive sciatic nerves are observed significantly more frequently in ovariectomized (OVX) rats than in sham rats (*P* = 0.035; Mann–Whitney U test).

Figure 2 shows representative images of pentosidine staining of the sciatic nerves from OVX (Figure 2A, 2B) and sham (Figure 2C, 2D) rats. Brown pentosidine staining in the nerve fibers of OVX rats was significantly increased compared to sham rats (*P* = 0.035; Figure 3).

Relationship between pain threshold and accumulation of pentosidine in the sciatic nerve

**Figure 4:**
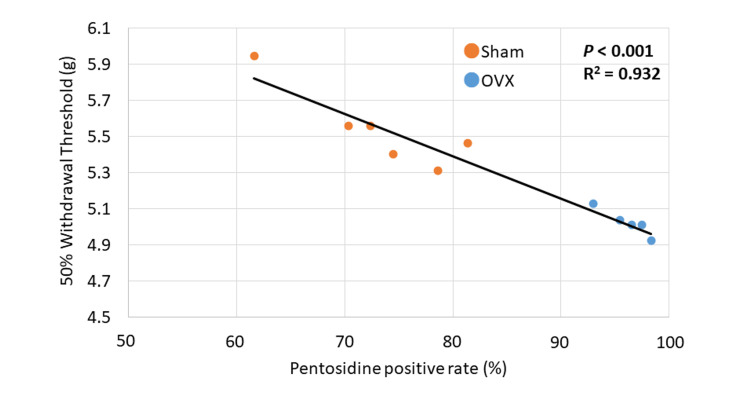
Relationship between withdrawal threshold and pentosidine positivity rate of sciatic nerve fibers The pentosidine positivity rate of sciatic nerve fibers shows a negative correlation with the withdrawal threshold (*P* < 0.001).

The pentosidine positivity rate of sciatic nerve fibers showed a negative correlation with the 50% pain threshold (*P* < 0.001; Figure 4). The data shows the same relationship, regardless of the group.

## Discussion

AGEs have recently attracted attention for their potential as biomarkers for clinical symptoms, in addition to aging. There are some reports about the mechanism of formation or accumulation of AGEs. For example, AGEs can increase in response to chronic high blood sugar and the glycation-related stress that accompanies it [5]. During diabetes, high concentrations of AGEs are formed, but AGEs can also form as part of aging. After AGEs are formed, it is difficult to reverse the process. Thus, AGE accumulation reflects a history of diabetes or other lifestyle-related diseases that are characterized by chronic high blood sugar, glycation stress, and oxidative stress. As a result, AGE accumulation leads to the degradation of bone and muscle function [9-12]. However, AGE patterns in orthopedic patients have not been clarified.

A relationship exists between oxidation and low levels of estrogen in postmenopausal women with osteoporosis [6], whereby estrogen inhibits oxidative modifications. This increase in oxidative stress leads to the accumulation of AGEs during menopause. Thus, in both patients with diabetes and dialysis and in menopausal osteoporotic patients, glycation and oxidative stress result in high levels of AGE formation.

In this study, sciatic nerves of ovariectomized rats exhibited higher pentosidine positivity rates than sham rats by pentosidine staining 8 weeks after ovariectomy. Based on this finding, we hypothesize that a lack of estrogen may cause the accumulation of AGEs at peripheral nerves.

OVX rats also exhibited plantar hypersensitivity to mechanical stimuli as compared to sham rats by von Frey test 8 weeks after ovariectomy. In the OVX mouse model of osteoporosis, a number of features have been reported: cutaneous hindpaw hypersensitivity, allodynia, and deep musculoskeletal pain [2]. The mechanisms underlying these findings are unclear, but some studies report a relationship between neurodegeneration and AGEs. AGEs are localized to peripheral nerves and AGE expression is increased following trauma or disease [13-15]. AGEs also affect sensory neurons as well as the cells that surround them, including Schwann cells, endothelial cells, smooth muscle cells, and monocytes or macrophages [16].

In the present study, pentosidine staining was observed in both axons and myelin sheaths. Previous reports indicated that pentosidine were identified in the neuronal perikarya and the extraneuroperikaryal deposits of both the Alzheimer’s disease and aged brain [17]. Peripheral nerve myelin has been reported to be modified by AGEs in diabetic neuropathy [18], but there has been no previous report describing the accumulation of pentosidine in peripheral nerve fibers in detail.

This study showed that the pentosidine positivity rate of sciatic nerve fibers was negatively correlated with the plantar mechanical hypersensitivity threshold in ovariectomized rats, suggesting that accumulation of pentosidine at nerves correlates with cutaneous hypersensitivity and might be associated with postmenopausal osteoporotic pain.

The present study had some limitations. First, we could not directly show the correlation between pentosidine positivity rate and von Frey test in this study. However, the ovariectomized rats showed significantly increased plantar hypersensitivity by von Frey test, compared to sham controls and pentosidine-positive sciatic nerves are observed significantly more frequently in ovariectomized rats. Therefore, we considered them to be indirectly related. Second, although the biological effects of ovariectomy may be significant, in this study we focused only on the accumulation of pentosidine at the sciatic nerve. We evaluated only the presence of the pentosidine at sciatic nerves and not quantitatively evaluated the accumulated pentosidine. We did not quantify the amount of pentosidine in blood and urine. Finally, we do not measure AGEs other than pentosidine.

## Conclusions

OVX rats showed significantly increased plantar hypersensitivity to mechanical stimuli compared to sham rats 8 weeks after ovariectomy. The sciatic nerve of ovariectomized rats showed a significantly higher pentosidine positivity rate than sham rats. The pentosidine positivity rate of sciatic nerve fibers was negatively correlated with withdrawal threshold, suggesting that the higher the level of pentosidine in sciatic nerve fibers, the more plantar hypersensitivity. Our results suggest that accumulation of pentosidine at the sciatic nerve, caused by ovariectomy, could lead to cutaneous hindpaw hypersensitivity, and this potential causal relationship needs further investigation.
